# RF Sputtering, Post-Annealing Treatment and Characterizations of ZnO (002) Thin Films on 3C-SiC (111)/Si (111) Substrates

**DOI:** 10.3390/mi8050148

**Published:** 2017-05-07

**Authors:** Visakh Valliyil Sasi, Abid Iqbal, Kien Chaik, Alan Iacopi, Faisal Mohd-Yasin

**Affiliations:** Queensland Micro- and Nanotechnology Centre, Griffith University, Nathan 4111, Australia; visakh.valliyilsasi@griffithuni.edu.au (V.V.S.); abid.iqbal@griffithuni.edu.au (Ab.I.); k.chaik@griffith.edu.au (K.C.); a.iacopi@griffith.edu.au (A.I.)

**Keywords:** ZnO, silicon carbide, radio frequency (RF) sputtering, annealing

## Abstract

We report on the radio frequency (RF) sputtering of c-axis oriented ZnO thin films on top of epitaxial 3C-SiC-on-Si (111) substrates, which were then subjected to post-annealing treatment at 400, 600 and 800 °C. Grazing incident X-ray Diffraction (XRD) data show that the Full Width Half Maximum (FWHM) values for O_2_/Ar ratios between 30% and 60% are consistent, with a mean of 0.325° and a standard deviation of 0.03°. This is largely attributed to the smaller lattice mismatch of 5% between the ZnO (002) and SiC (111) films. The quality of the ZnO films deteriorated at the post-annealing treatment of 800 °C, as demonstrated by the increasing value of FWHM diffraction peaks, the reducing value of the peak intensity, the reducing percentage of (002) oriented area under the curve, and the increasing value of biaxial stress. We propose a simple growth model to explain the result.

## 1. Introduction

Zinc oxide (ZnO) is a group II-IV semiconductors, which is suitable for a wide range of scientific and technological applications. It has a wide band gap of 3.3 eV and an excitation binding energy of 60 meV at room temperature [1]. ZnO can potentially be used in solar cells, gas sensors and optical wave guides because of its excellent electrical and optoelectronic properties [2]. It can also be used in the fabrication of surface acoustic wave (SAW) devices, piezoelectric transducers and film bulk acoustic resonators [3]. ZnO is commonly deposited via a sputtering system because this requires a relatively simple apparatus, low substrate temperature, high deposition rate, good surface flatness, high transparency and dense layer formation [4].

ZnO thin films are normally deposited on silicon (Si) substrates because of the ease in fabrication and the low cost per unit surface area. However, the large difference of 39% in lattice mismatch and 68% in thermal expansion coefficients (CTE) generates cracks, dislocations and stresses in deposited films that ultimately results in the deterioration of ZnO crystal quality [5]. Therefore, ZnO films have been deposited on various substrates with lower CTE and lattice mismatches e.g., α-Al_2_O_3_ [6], GaN [7], AlN [8], 3C-SiC [9], 4H-SiC [10] and 6H-SiC [11]. There were two groups that have performed radio frequency (RF) sputtering of ZnO (002) on top of 3C-SiC-on-Si substrates, similar to our work. Sha et al. compared the Full Wave Half Maximum (FWHM) values of the diffraction peaks of ZnO (002) that were RF sputtered on top of Si (111) and 3C-SiC-on-Si (111) substrates [12]. They observed FWHM values of 0.65° to 0.55° on both substrates, respectively. They further annealed the samples at 1000 °C, and demonstrated the reduction in the FWHM values of both samples down to 0.19° and 0.25°, respectively. However, all of their samples were polycrystalline with no preferred orientation. Phan et al. compared the crystal quality of ZnO (002) deposited on top of 3C-SiC (111)/Si (100) substrates using RF sputtering and sol-gel [5]. The 3C-SiC buffer layer was a polycrystalline with (111) orientation. They observed a dominant (002) orientation using RF sputtering. They reported FWHM diffraction peak values of 0.2107° and 0.3498°, respectively, for both methods at the annealing temperature of 800 °C. In a subsequent work from the same group [13], they fabricated ZnO-based SAW resonators on top of Si (100) and 3C-SiC/Si (100) substrates. They reported improved insertion loss and temperature stability of the resonator made on top of the 3C-SiC (111) buffer layer. Both Sha et al. and Phan et al. demonstrated that ZnO thin film with dominant (002) orientation could be sputtered on top of Si substrate with a polycrystalline 3C-SiC buffer layer.

In this paper, we performed RF sputtering to obtain *c*-axis oriented ZnO films on top of Si (111) substrates with 3C-SiC (111) epitaxial buffer layer. Specifically, we studied the effect of varying the O_2_/Ar ratios on the ZnO (002) crystal orientation, and found that the FWHM values are consistent between 30% to 60% O_2_/Ar ratios. We illustrated the effect of 5% lattice mismatch between ZnO (002) and 3C-SiC (111) buffer layer that contributed to this interesting result. We further subjected the samples to post-annealing treatment at three temperatures, and proposed the growth model for the optimized annealing temperature of 600 °C. It must be noted that the samples for this article were batch-fabricated and batch-tested together with ZnO (002) films on top of 3C-SiC-on-Si (100) substrates. The results for the latter were recently reported in [14].

## 2. Experimental Methods

The deposition of ZnO films was performed on top of a 3C-SiC-on-Si substrate using an RF sputterer (Denton Vacuum, Moorestown, NJ, USA) operating at 450 W. It is a common sputtering technique to employ the maximum available power to obtain the highest deposition rate and best crystal quality. Since the maximum power of the RF sputterer is 500 W, we chose 450 W with a 50 W gap from the maximum power to avoid damaging the equipment. Prior to this work, the 3C-SiC (111) thin film with a thickness of 300 nm was epitaxially grown on top of Si (111) wafer with a diameter of 150 mm using a custom-made hot-wall horizontal low pressure chemical vapour deposition (LPCVD) system at Queensland Micro- and Nanotechnology Centre of Griffith University [15]. Our center has been producing epitaxial 3C-SiC/Si wafers of different 3C-SiC thicknesses (ranging from 50 nm to 2 µm) and wafer sizes (ranging from 2 to 12 inches in diameter). The 300-nm thick 3C-SiC layer was arbitrarily chosen during the time of this experiment. In the first experiment, the samples were cut in a size of 15 × 15 mm^2^ using a wafer dicer (Disco Corp., Tokyo, Japan) and cleaned via the standard piranha cleaning process to remove organic substances. The sputtering system was turbo-pumped for 15 min to the base pressure of 5 × 10^−5^ Torr to reduce residual gasses in the chamber and hence eliminate unwanted reactions. The sputtering pressure of 4 mTorr was used for all the depositions. The substrate temperature of 200 °C was chosen to follow industry practice. The Zinc (Zn) target of 100 mm in diameter and 6.35 mm in thickness with 99.999% purity was employed. Argon gas was pumped to the chamber for 6 min with the substrate’s shutter closed in order to clean the Zn target from previous reactions. The distance from the target to the substrate was 76 mm, which is a default distance of the RF sputterer. The O_2_/Ar ratio was increased from 30% to 60% in a step of 10% with a total gas flow of 34 sccm to evaluate the effect of O_2_ concentration on the crystal orientation. The poison mode for the ZnO was determined by measuring the conductivity of the films, since ZnO is highly insulative. The deposition time was 30 min. After the deposition, it took 90 min for the substrate temperature to cool down to 50 °C before the samples were taken out of the chamber. The deposition rate was determined by measuring the thickness of the ZnO films using a step profiler (Dektak stylus profilometer, Bruker, Billerica, MA, USA) over the total deposition time.

The second experiment was to subject the samples to post-annealing treatment. This step was needed to improve the crystal quality of ZnO (002) as demonstrated by the systematic studies of annealing ZnO films on Si substrates conducted by Fang et al. [16]. Our samples were placed into the annealing chamber (custom-made glass tube). Nitrogen gas was pumped into the chamber with a flow of 1700 cc/min. We annealed our samples at three different temperatures i.e., 400, 600 and 800 °C. The samples were then cooled down to 50 °C before removing them from the chamber. The cooling rate is non-linear and the duration depends on the highest temperature in the chamber. For the annealing temperature of 400 °C, it took 60 min to cool down to 50 °C. Therefore, the average cooling rate is 6 °C/min.

The deposited ZnO films were characterized by the following parameters (equipment): X-ray diffraction (D8 Advance X-Ray diffraction tool using monochromatorized Cu Kα1 beam with λ = 1.5405980 Å, Bruker), surface roughness (tapping-mode Cypher Atomic Force Microscopy with etalon cantilever, Asylum Research, Santa Barbara, CA, USA) and film thickness (Dektak stylus profilometer, Bruker).

## 3. Results and Discussion

**Figure 1:**
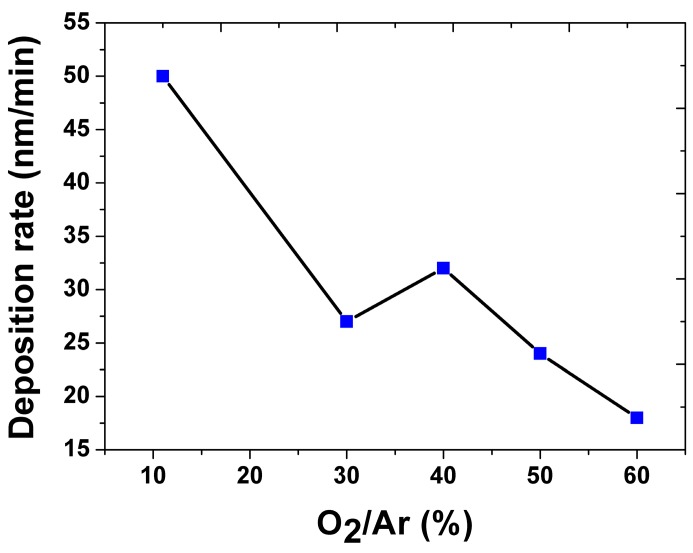
Deposition rate versus O_2_/Ar ratio.

The deposition rate at different O_2_/Ar ratios is shown in Figure 1. The general trend is that the deposition rate decreases with the increase in O_2_ concentration. The increase in O_2_ atoms decreases the concentration of the heavier Ar atoms that negatively affect the deposition rate. The only anomaly is the increasing deposition rate from 30% to 40% O_2_/Ar ratio. It seems that a rapid oxidation of the target took place between these two O_2_ concentrations. The oxide targets have a higher secondary electron yield compared to the metal targets, which causes more ionization of the sputtering gas and an increase in the deposition rate. A further increase in the oxygen concentration forms a surface layer of adsorbed oxygen, which prevents the sputtering of the atoms and thus reduces the deposition rate. Also, the concentration of the heavier Ar atoms decreases with an increase in O_2_, so the net deposition rate decreases afterwards [17].

**Figure 2:**
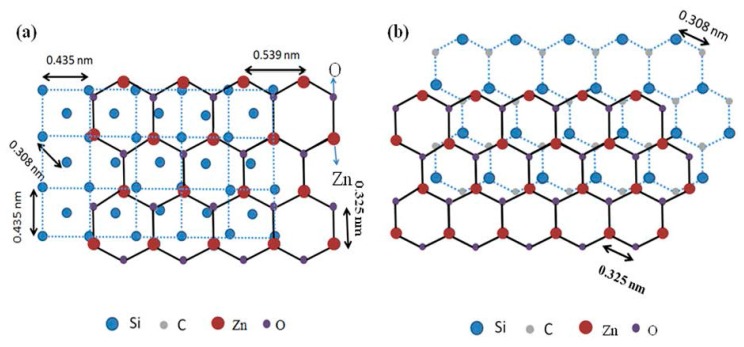
Crystal structures of (**a**) ZnO (002) on 3C-SiC (100); and (**b**) ZnO (002) on 3C-SiC (111).

The crystallinity of the deposited films depends on the quality of the nucleation layer and the nature of the bonding between the substrate and deposited film [18]. The crystal structure of the c-axis oriented ZnO superimposed over the 3C-SiC (100) and 3C-SiC (111) crystal structure [19] is shown in Figure 2a,b respectively. The 3C-SiC (111) has a six-fold atomic arrangement (three-fold symmetry) that provides a better template and lattice matching for the *c*-axis oriented ZnO. Also, the smaller lattice mismatch (5.5%) between 3C-SiC (111) and ZnO (002), as opposed to the 33.8% lattice mismatch between 3C-SiC (100) and ZnO (002), provides a better template for *c*-axis oriented ZnO film growth.

At the beginning of sputtering, the ZnO seed layer is grown along the (002) orientation via the creation of bonds with the three-fold symmetrical hexagonal 3C-SiC (111) substrate. The ZnO layer follows the seed layer for the rest of sputtering and grows in well-aligned manner on the seed layer in a close-packed (002) orientation. It will be demonstrated later that this resulted in the consistent crystal quality of ZnO (002) across a wide range of O_2_/Ar ratios.

Table 1 shows the summary of the obtained data, which can be divided into three parts. The first part shows the characteristics of the two samples at 40% and 60% O_2_/Ar ratios, which were not subjected to the post-annealing treatment. The second part summarizes the characteristics of the samples that were subjected to the post-annealing treatment at three different temperatures. The third part details the characteristics of the annealed samples of 600 °C at four different O_2_/Ar ratios. The following parameters, namely, peak position for (002) orientation, percentage of the area under the curve for (002) orientation, FWHM of the diffraction peak of (002), and peak intensity (002) are extracted from the XRD plots of the samples. We extracted the values of the FWHM of the diffraction peaks using Gaussian fitting. The grazing incident X-ray diffraction (GIXRD) measurement was employed due to the smaller thickness of the ZnO films compared to the substrates. The grain size was calculated from the FWHM of (002) the diffraction peak by the Sherrer equation [20]. The biaxial stress (*σ*) was calculated using the following equation: *σ* = −453.6 × 10^9^((*c* − *c*_0_)/*c*_0_), where *c*_0_ is the strain-free lattice parameter (*c*_0_ *=* 5.205 Å) measured from a ZnO powder sample [21]. The value of *c* was extracted from the experimental data as follows: *c* = 2*d*, where *d* = *λ*/2 × sin *θ* from Bragg’s law, where *λ* = 1.5405980 Å, and *θ* is the diffraction angle from the XRD 2θ plot.

Several important observations can be deduced from Table 1. First, the effect of post-annealing on the FWHM of the diffraction peaks and the grain size are highly visible. For example, the FWHM value of the samples that were sputtered using a 40% O_2_/Ar ratio is reduced from 0.465° to 0.31°. Their grain size increases from 18.8 to 28.04 nm. A similar trend can be seen for the samples at a 60% O_2_/Ar ratio. Annealing increased the energy of film atoms thus enhancing the adatoms’ mobility, which in turn decreased the defects in the ZnO films and improved the quality of films. The small crystallites coalesced together at a high temperature to form larger crystallites when they are annealed, which increases the grain size, corroborating the reduction in the FWHM of the diffraction peaks [16].

**Figure 3:**
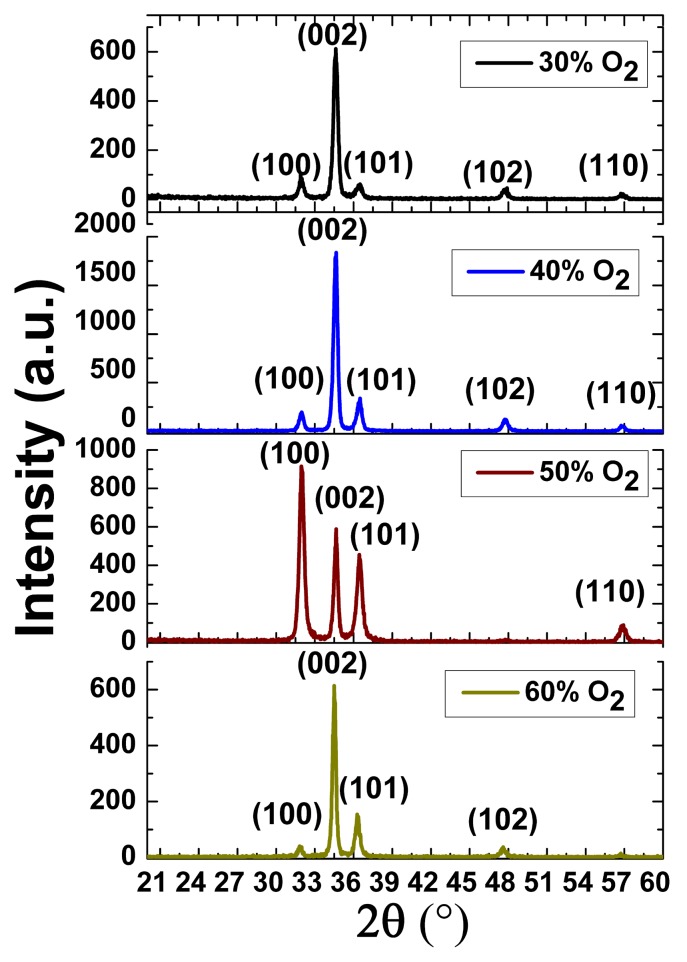
Grazing angle XRD at 30%, 40%, 50% and 60% O_2_/Ar ratios at the annealing temperature of 600 °C.

The second observation is in terms of the effect of different O_2_/Ar ratios on the (002) crystal orientation, after annealing the samples at 600 °C. The (002) orientation dominates between 30% to 60% O_2_/Ar ratio. The only exception is at a 50% ratio, where the quality of ZnO (002) film may have been influenced by the lower quality of the specific substrate that was diced from the 3C-SiC (111)/Si (111) wafer. Figure 3 shows the θ-2θ scan for the samples prepared at 30%, 40%, 50% and 60% O_2_/Ar ratios. The GIXRD measurement had the following parameters. The voltage and current used for all measurements was 40 kV and 40 mA. The 2*θ* range was ~25 to 60 degrees and the glancing angle was 1°. The step size was 0.04°, and the time per step was 0.9999 s. The GIXRD spectra shows three major diffraction peaks at 2*θ* = 32, 34 and 37°, which correspond to (100), (002) and (101) crystal orientations of ZnO (JCPDS card number 01-089-1397), respectively. Two minor diffraction peaks are also observed at 2θ = 47 and 57°, which correspond to (102) and (110) ZnO crystal orientations, respectively. We extracted the FWHM of the diffraction peak of (002) from Figure 2. The FWHM values and the area of (002) under the curve for these different O_2_/Ar ratios were very similar, with a mean of 0.325° and a standard deviation of 0.03°. Previously, we reported that we had been unsuccessful in depositing ZnO (002) films on top of 3C-SiC (100)/Si (100) substrates at 60% O_2_/Ar ratios [14]. The fact that we have been able to do that for this experiment can be attributed to the smaller lattice mismatch of 5% between ZnO and SiC (111) [9]. Similar observations for AlN (002) film depositions have been reported by Lim et al. [22]. It is evidenced from these data that the ZnO (002) orientation can be formed on top of Si substrates with 3C-SiC buffer layer in the range of 30% to 60% O_2_/Ar ratios.

**Figure 4:**
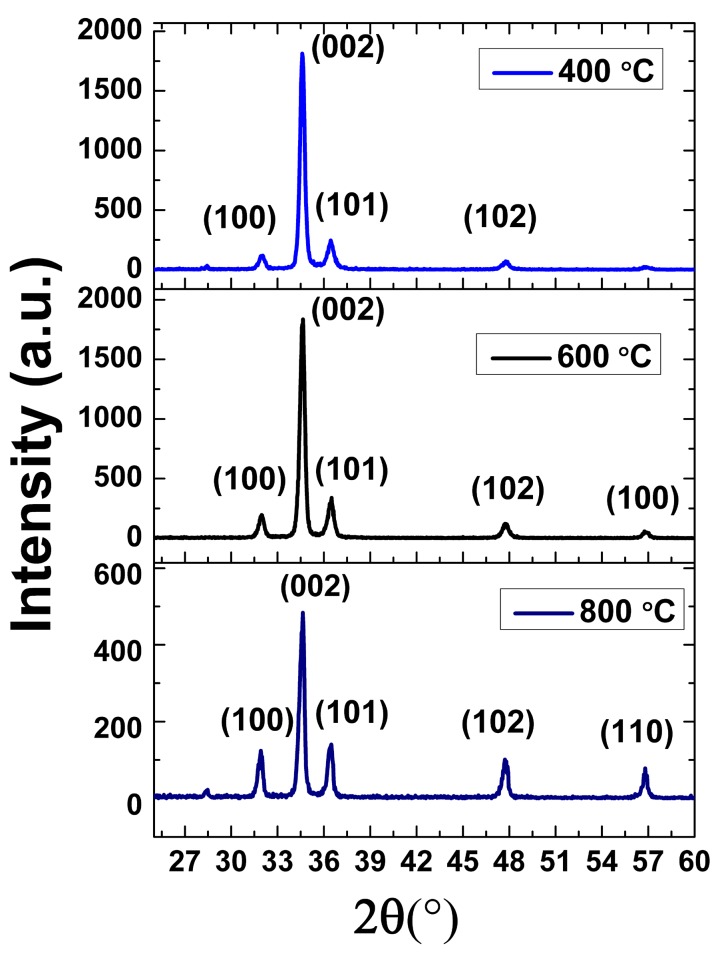
Grazing angle XRD of annealed samples at a 40% O_2_/Ar ratio. These samples are annealed at 400, 600 and 800 °C.

The third observation is in terms of the effect of different annealing temperatures (400, 600 and 800 °C) on the (002) crystal orientation at a 40% O_2_/Ar ratio. Figure 4 shows the GIXRD spectra for these samples. The major diffraction peak depicting (002) orientation is prominent in all the samples. The FWHM of the diffraction peaks of (002) orientation were calculated as 0.33°, 0.31° and 0.35° at 400, 600 and 800 °C, respectively.

**Figure 5:**
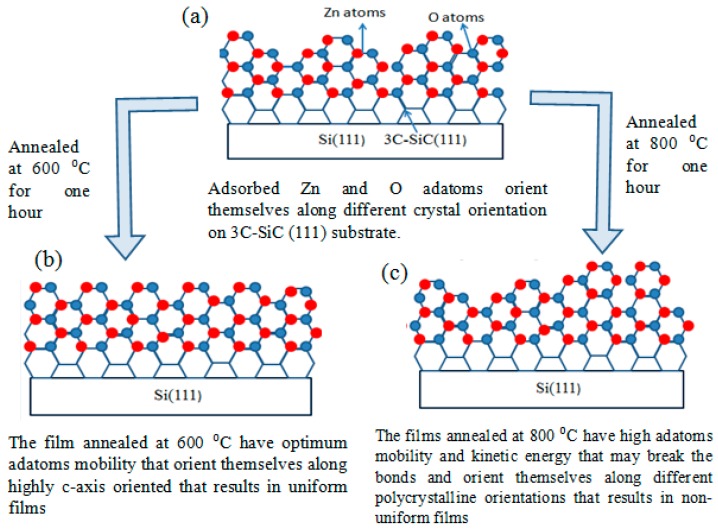
(**a**) Growth model of the ZnO film on top of the 3C-SiC (111)/Si (111) substrate prior to the post-annealing treatment. (**b**) Growth model of the ZnO film on top of the 3C-SiC (111)/Si (111) substrate after the post annealing treatment at 600 °C. (**c**) Growth model of the ZnO film on top of the 3C-SiC (111)/Si (111) substrate after the post annealing treatment at 800 °C.

It is evident from Figure 4 that 600 °C is the optimized annealing temperature, that provides the optimum adatoms mobility to orient themselves along the *c*-axis for the deposition of ZnO (002) orientation on top of 3C-Si (111) substrates. The GIXRD scan reveals that the crystal quality seems to deteriorate at 800 °C, as demonstrated by the FWHM values, as well as the reducing value of the peak intensity, the reducing percentage of (002) oriented area under the curve, and the increasing value of biaxial stress. Fang et al. [16] noticed the same trend for the annealing of ZnO (002) on top of Si (100) and attributed it to the presence of porosity. Since the grain sizes remain consistent at different annealing temperatures, as shown in Table 1, this theory is not applicable in our case. Singh et al. offered another hypothesis [23], that is, that the quality of ZnO film deteriorates at higher annealing temperatures due to the breakage of bonds and/or reaction with the substrate. We explore the breakage bond theory and illustrate its effect in Figure 5. The film that was annealed at 600 °C has optimized kinetic energy to orient the adatoms along the (002) orientation. In the case of the film that was annealed at 800 °C, the adatoms had higher kinetic energy and surface mobility that could have broken the bonds. As a result, in addition to (002) orientation, the highly energetic adatoms also orient themselves along other crystal orientations, such as (100), (101), (102) and (110).

**Figure 6:**
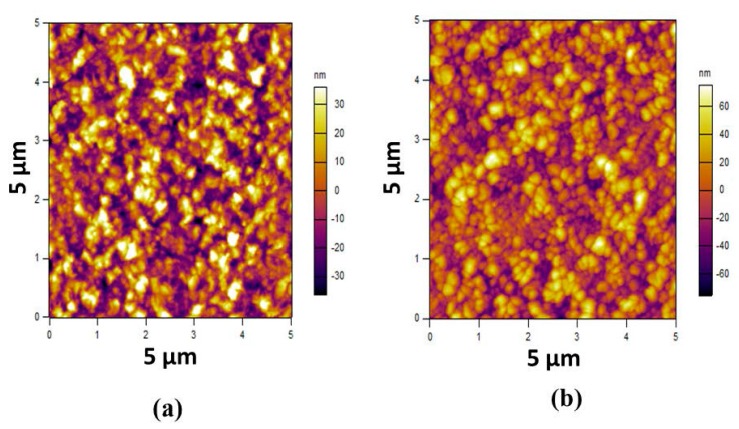
Atomic Force Microscopy (AFM) images of ZnO of (**a**) un-annealed ZnO/3C-SiC (111)/Si (111); and (**b**) annealed ZnO/3C-SiC (111)/Si (111) samples at 40% O_2_/Ar concentrations.

The Atomic Force Microscopy (AFM) scan of the un-annealed and annealed (at 600 °C) samples prepared at a 40% O_2_/Ar ratio are shown in Figure 6. The root mean square (rms) roughness of both deposited films was measured in a 5 μm  ×  5  μm area using tapping mode Atomic Force Microscopy (Cypher AFM) with an etalon cantilever. The rms value is calculated using Igor software. The roughness of the un-annealed sample is 16.5 nm (in rms), which increased to 24 nm (in rms) after post-annealing treatment at 600 °C. The increase in roughness is due to recrystallization at a high temperature, in which small crystallites coalesce together to make larger crystallites [24].

## 4. Conclusions

We have demonstrated the RF sputtering of *c*-axis oriented ZnO thin films on top of epitaxial 3C-SiC-on-Si (111) substrates. Four key results are highlighted. First, the value of the FWHM of the (002) diffraction peak was reduced by a factor of 50% after post-annealing, illustrating its effectiveness. Second, after the post-annealing treatment, the FWHM values of ZnO (002) for the samples from 30% to 60% O_2_/Ar ratios showed minimal changes, with the mean of 0.325° and standard deviation of 0.03°. Third, the post-annealing temperature of 600 °C was found to be the most optimized range for the ZnO (002) deposition. This is supported by the data from the extracted FWHM and the value of the biaxial stress. We have also proposed a growth model to explain the effect of the post-annealing treatment on the crystal quality of the ZnO films. Fourth, the surface roughness of the ZnO film increased after annealing due to the coalescence of small crystallites. This observation is supported by the AFM measurement and is corroborated by the calculation of the grain size.

## Figures and Tables

**Table 1:** Summary of data for ZnO (002).

Experiment	Sample ID	O_2_/Ar Ratio (%)	Annealing Temperature (°C)	Peak Position (002)	Area for (002) (%)	Thickness (nm)	FWHM of Diffraction Curve (°)	Peak Intensity (cps)	Grain Size (nm)	Biaxial Stress (GPa)
No annealing	V10	40	-	34.23	60	960	0.465	540	18.8	2.58
	V9	60	-	34.26	98	540	0.59	780	14.63	2.194
Effect of Annealing Temperature	C7	40	400	34.63	70	960	0.31	1750	28.04	2.178
	VA3	40	600	34.62	71	960	0.32	1836	27.16	2.52
	C3	40	800	34.64	53.6	960	0.33	484	26.18	2.788
Effect of O_2_ Ratio (annealed)	VA1	30	600	34.6	73.7	810	0.32	578	27.1	2.655
	VA3	40	600	34.62	71	960	0.32	1836	27.16	2.52
	VA5	50	600	34.65	22	720	0.37	506	23.5	2.78
	VA10	60	600	34.55	68	540	0.29	561	29.98	1.51
